# Unilateral Hypoplasia of the Trapezius Muscle in a Female Cadaver

**DOI:** 10.7759/cureus.98080

**Published:** 2025-11-29

**Authors:** Alyssa Levien, Avery M Savoie, Kelsey Negron, David Jaynes

**Affiliations:** 1 Department of Anatomical Sciences, Edward Via College of Osteopathic Medicine, Spartanburg, USA

**Keywords:** anatomical variations, congenital, hypertrophy, muscle agenesis, rhomboid muscle

## Abstract

Dissection of a 67-year-old female cadaver revealed a left-sided trapezius muscle of diminutive size. An examination of nerve supply and vasculature yielded no apparent anomalies, which suggests a developmental etiology. Earlier reports suggest that the trapezius is likely derived from at least two differing cell populations. It is possible that one or both failed to contribute fully to the development of the trapezius muscle in this individual. The rhomboid muscles, which share some functions with the trapezius muscles, demonstrated significant ipsilateral compensatory hypertrophy, likely due to increased use. Although varying abnormalities of the trapezius have been previously observed, this case displayed unique attachment points for a trapezius muscle with congenital hypoplasia. When considered alongside the other reported cases of varying trapezius hypoplasia, this case highlights that trapezius anomalies should be an important differential diagnosis in patients with chronic upper limb weakness and back asymmetry.

## Introduction

The trapezius muscles collectively form a trapezoidal superficial posterior axio-appendicular muscle that provides direct attachment of the pectoral girdle to the vertebral column and cranium. The motor component of the innervation to the trapezius muscle is through the spinal accessory nerve (CN XI), and the sensory fibers are derived from C3-C4 ventral rami. The trapezius muscle derives its primary vascular supply from the transverse cervical artery [1]. 

The trapezius muscle arises from the superior nuchal line, external occipital protuberance, ligamentum nuchae, and spinous processes of C7-T12 vertebrae. It is divided into three parts based on fiber orientation and attachments, each of which imposes different actions on the scapula. The superior portion, composed of descending muscle fibers, originates at the external occipital protuberance, the medial third of the superior nuchal line, and the nuchal ligament. It inserts on the clavicle to elevate the scapula and aid in rotation, flexion, and extension of the head and neck. The middle part (transverse fibers) originates on the spinous processes of T1-T4, inserts on the spine of the scapula, and functions to draw the scapula medially. The inferior component consists of ascending fibers that originate on the spinous processes of vertebrae T4-T12 and insert on the medial end of the scapular spine; the main action is to draw the scapula inferomedially [1]. As a whole, the trapezius muscle plays a crucial role in stabilizing the scapula during everyday activities that involve shoulder elevation and depression, rotation of the head, and movements of the upper limb.

In this particular case, the left trapezius muscle demonstrated atypical morphology characterized by altered attachment points, attributable to partial agenesis of its superior and inferior components. Consequently, the overall size of the muscle was reduced. Although the attachment points were unique, the vasculature and innervation of the trapezius muscle were observed to be normal. 

In addition to hypoplasia of the left trapezius muscle, the ipsilateral rhomboid muscles were noticeably larger in size than those on the right. The major and minor rhomboid muscles lie deep to the trapezius and form parallel bands that pass inferolaterally from the C7-T5 vertebrae to the medial border of the scapula. Both muscles are innervated by the dorsal scapular nerve and supplied by the dorsal scapular artery. Similar to the trapezius, the rhomboids assist in holding the scapula against the thoracic wall, fix the scapula during upper limb movement, and assist in retraction of the scapula [1]. 

When a muscle is reduced in size and therefore generates reduced force, the stability of the surrounding musculoskeletal structures is altered [2]. In this case of unilateral trapezius hypoplasia, scapular stability was likely compromised, resulting in asymmetry of the back and limited range of motion. These musculoskeletal abnormalities can cause individuals to experience pain and functional limitations, emphasizing the importance of recognizing trapezius anomalies in clinical evaluation, particularly in patients with Poland's syndrome, Sprengel's deformity, or other disorders that alter the axio-appendicular musculature. 

## Case presentation

**Figure 1:**
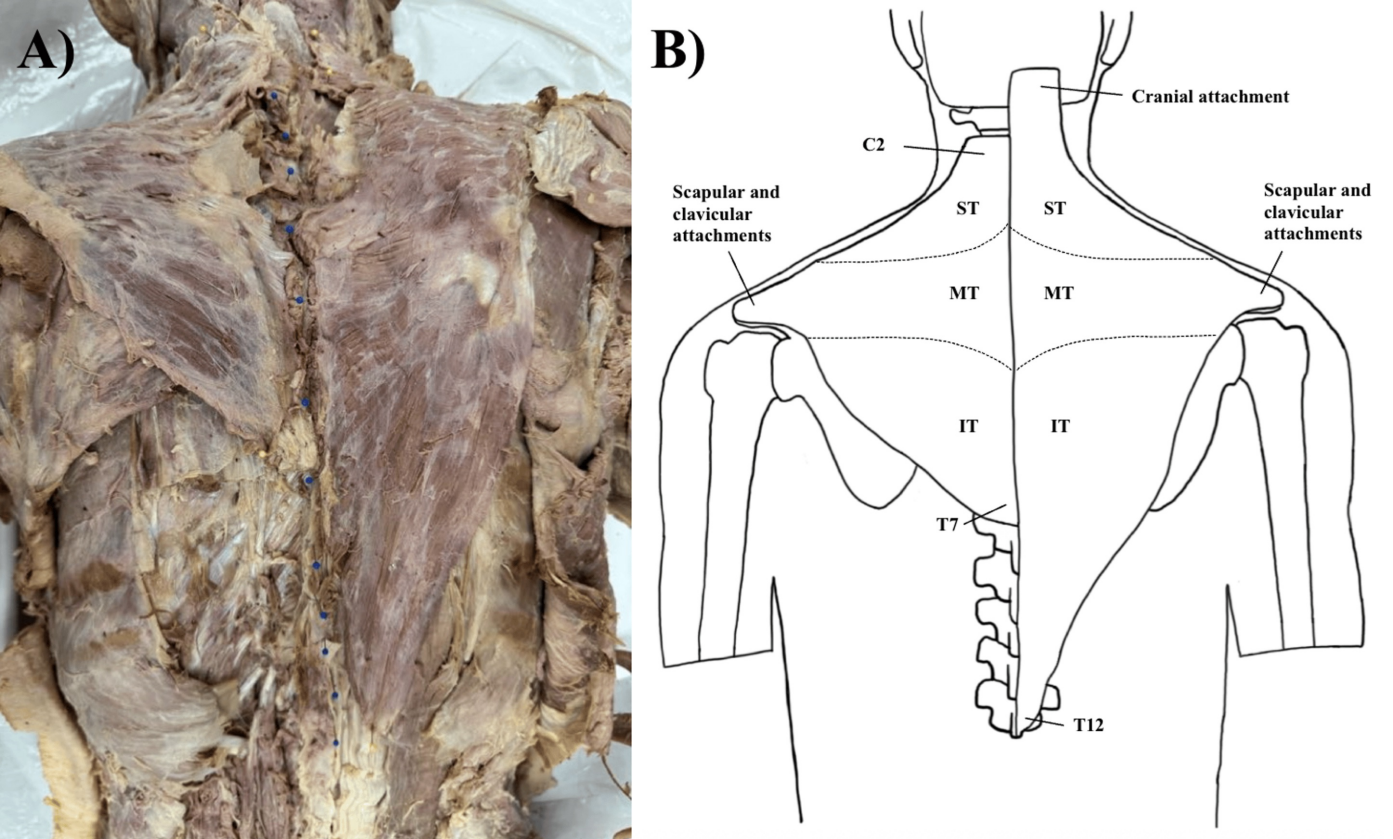
A) Dorsal view of the trapezius muscles. A)The blue pins represent the spinous process of each vertebra. This was done to assess potential scoliosis that may have occurred as a result of a greater contralateral force. B) Accompanying illustration with labelled attachment points, created by the authors. ST: superior trapezius muscle,  MT: middle trapezius muscle, IT: inferior trapezius muscle.

A 67-year-old female cadaver was gifted to the anatomy program at the Edward Via College of Osteopathic Medicine-Carolinas Campus for medical education and anatomical research. During routine dissection, hypoplasia of the left trapezius muscle was observed. The superior part of the trapezius muscle only extended as far superiorly as C2, with no cranial attachments. The inferior part originated at the spinous process of T7 (Figure 1). The clavicular, acromial, and scapular insertions were normal, and there were no signs of previous trauma or surgical procedures. Additionally, a thorough investigation revealed typical innervation by the spinal accessory nerve and vascular supply from the transverse cervical artery. Hypoplasia of the trapezius muscle was only noted on the cadaver’s left side, as the right trapezius muscle displayed typical fiber orientation and attachment points. 

The fascia was cleaned from each trapezius muscle, which were then removed by cutting along their attachment points, leaving the aponeurosis intact. While the extent of aponeurosis varies, its entirety was retained in the excision of the muscle. Using a digital gram scale (Sigma-Aldrich, Inc., Missouri, USA; Accuris Mini), each muscle was weighed in grams. The left trapezius muscle weighed 92.71 g while the right trapezius muscle weighed 149.63 g. The anomalous trapezius was 62% reduced compared to the contralateral normal trapezius muscle (Table 1).

**Table 1:** Muscle weights in grams and accompanying comparative analysis.

	Left Side	Right Side	Difference
Trapezius muscle	92.71	149.63	0.62
Rhomboid major and minor muscles	70.70	42.25	1.67

**Figure 2:**
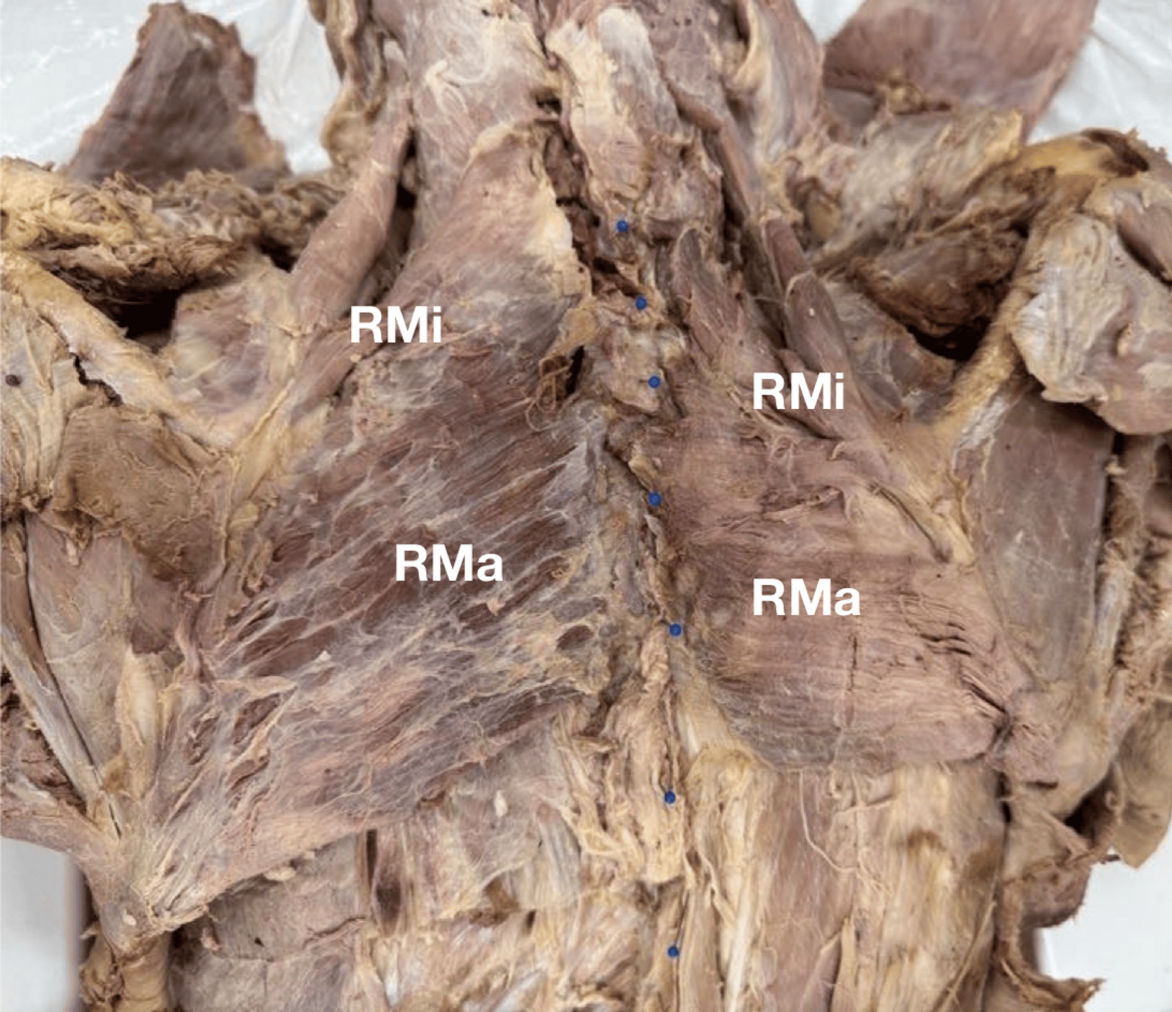
Cadaveric dissection of the superficial muscles of the back with the trapezius muscles reflected, revealing the rhomboid major and minor muscles. Note the considerable size disparity between the right and left rhomboid muscles. RMa: rhomboid major muscle, RMi: rhomboid minor muscle.

Upon reflecting the hypoplastic trapezius, the presence of considerably enlarged rhomboid major and minor muscles on the left side was observed (Figure 2). On both sides, the rhomboids maintained normal attachment points, innervation, and vasculature. The rhomboid muscles were collectively removed and weighed following the same method as the trapezius muscles. The left rhomboids weighed 70.70 g and the right rhomboids weighed 42.25 g (Table 1), indicating compensatory hypertrophy of these muscles. The other muscles in this region, for example, the pectoralis major, serratus anterior, latissimus dorsi, and supraspinatus muscles, exhibited no noticeable differences between sides.

## Discussion

Congenital anomalies of the musculoskeletal system are not uncommon; however, isolated developmental abnormalities of the trapezius muscle have not been frequently observed. The clinical presentation of Poland’s Syndrome includes anterior musculoskeletal abnormalities, such as absence of the costosternal portion of the pectoralis major muscle, complete absence of the pectoralis minor muscle, hypoplasia of breast or nipple tissue, subcutaneous fat deficiencies, and deformities of some of the costal cartilages. Some cases of Poland’s Syndrome have also been described as congenital absence or hypoplasia of the trapezius muscle [3]. This cadaver, however, did not present with Poland’s Syndrome. 

Unilateral agenesis of the trapezius muscle has been documented in association with Sprengel’s deformity, which is a congenital anomaly characterized by a hypoplastic, superiorly displaced scapula. This is thought to be due to disruption of the embryonic subclavian vascular supply [4]. The anatomical findings in this cadaver were not consistent with Sprengel’s deformity, as the scapulae were symmetrical in size and position.

Previous cases documenting hypoplasia of the trapezius muscle have also reported absence or hypoplasia of the sternocleidomastoid muscles [5]. Our case presented with typical attachment points, innervation, and vasculature for the sternocleidomastoid muscles. Other studies have reported unilateral trapezius hypoplasia in both live patients and cadaveric dissections [6,7]. Most cases reported hypoplasia limited to the superior portion of the muscle. Two studies have reported hypoplasia of the inferior portion of the trapezius muscle; both cases reported hypoplastic muscle fibers embedded within an aponeurosis, which was not observed in this cadaver [8]. One of these studies also proposed that unilateral hypoplasia of the trapezius may result in scoliosis in the region of the absent portion [9]. We evaluated this possibility and found that in our case, there was no detectable scoliosis. To our knowledge, this is the first case describing unilateral trapezius hypoplasia originating above the spinous process of T8, at the ligamentum nuchae of C2, and having no cranial attachment. Compensatory hypertrophy of the ipsilateral rhomboid muscles in a case of unilateral hypoplasia of the trapezius muscle has also not been previously reported. 

Observation of an intact spinal accessory nerve and typical blood supply suggests an embryologic etiology of the hypoplastic trapezius. The embryonic origin of the trapezius muscle has long been a subject of debate. Differing approaches employed to answer this question have consistently revealed the following: migratory post-otic neural crest (PONC) initiate myogenesis by establishing trapezius attachment sites located along the clavicle, acromion, and scapular spine and by forming the trapezius aponeurosis which acts as a scaffold for a separate, distinct population of cells (specifically, occipital lateral plate mesoderm) to infiltrate the area and differentiate into muscle fibers [10-12]. Although studies indicate that the connective tissue components associated with the trapezius are derived from PONC, there is evidence that a detectable percentage of these cells may differentiate into muscle fibers (the primary domain of occipital lateral plate mesoderm) [13]. It is possible that the environment occupied by either/both cell types, temporally and spatially, induces differentiation of disparate myogenic populations by activating transcription factors that upregulate or downregulate myogenic regulatory factors e.g., myogenic factor (MYF) and myogenic differentiation (MYOD), [14-16]. Studies have shown that several tissues, such as surface ectoderm, paraxial mesoderm, the notochord, and the neural tube, secrete unique molecules that locally impact the differentiative pathway taken by precursor cells [17,18]. That there are at least two cell types working collaboratively to form the trapezius may, in part, explain the various degrees of trapezius aplasia that have been reported, as well as the variable part of the trapezius that is deficient. It is also a possibility that the muscle formed normally in the embryo, but then the absent portion degenerated.

Skeletal muscle is highly adaptable, and muscular hypertrophy has been shown to be a morphological response to functional reduction of synergistic muscles in an effort to maintain some degree of function. Compensatory hypertrophy of the left rhomboid muscles was visible to the naked eye when the trapezius muscles were reflected. The left rhomboids were noted to be 1.67 times larger by weight than the right rhomboid muscles (Table 1). The unilateral hypertrophy could have placed unequal stress on the rhomboid muscles’ attachment points, including the spinous processes of C7-T5 and the medial border of the scapula, which might have resulted in gross asymmetry, clinical muscular imbalances, and pain in this individual. 

While the extent of strength and movement loss in this individual can only be speculated, it likely included weakness in actions dependent on trapezius function, such as leftward rotation of the head, elevation and depression of the left shoulder, and internal rotation of the left upper limb. Live patients with other variations of unilateral trapezius hypoplasia have also displayed scapular dyskinesis [19]. Further, there are surgical procedures such as the lower trapezius tendon transfer, in which the tendon of the inferior portion of the trapezius is used to replace otherwise irreparable rotator cuffs [20]. This operation and other similar surgeries would be hindered in persons exhibiting trapezius hypoplasia. 

## Conclusions

To the best of our knowledge, this is the first case of unilateral hypoplasia of a trapezius muscle with no cranial attachment and the inferior part originating at the spinous process of T7. This is also the first case with compensatory hypertrophy of the ipsilateral rhomboid muscles. This report aims to serve as a resource for clinicians to aid in the recognition of anatomical variations in clinical settings and to acknowledge the possible impact hypoplastic trapezius muscles can have on the structure and function of the upper extremity, as well as the other synergistic axio-appendicular muscles. Further research into the embryological development of the trapezius muscle may help to clarify the causes of congenital anomalies like the one described here.
